# Blocking Connexin-43 mediated hemichannel activity protects against early tubular injury in experimental chronic kidney disease

**DOI:** 10.1186/s12964-020-00558-1

**Published:** 2020-05-25

**Authors:** Gareth W. Price, Christos E. Chadjichristos, Panagiotis Kavvadas, Sydney C. W. Tang, Wai Han Yiu, Colin R. Green, Joe A. Potter, Eleftherios Siamantouras, Paul E. Squires, Claire E. Hills

**Affiliations:** 1 Joseph Banks Laboratories, School of Life Sciences, University of Lincoln, Green Lane, Lincoln, UK; 2 National Institutes for Health and Medical Research Unite Mixte de Recherche S1155, Batiment Recherche, Tenon Hospital, 4 rue de la Chine, 75020 Paris, France; 3 Division of Nephrology, Department of Medicine, The University of Hong Kong, Hong Kong, Hong Kong; 4 Department of Ophthalmology, New Zealand National Eye Centre, University of Auckland, Auckland, New Zealand

**Keywords:** Chronic kidney disease, Connexins, Hemichannels, ATP, Cell adhesion, Tubular injury

## Abstract

**Background:**

Tubulointerstitial fibrosis represents the key underlying pathology of Chronic Kidney Disease (CKD), yet treatment options remain limited. In this study, we investigated the role of connexin43 (Cx43) hemichannel-mediated adenosine triphosphate (ATP) release in purinergic-mediated disassembly of adherens and tight junction complexes in early tubular injury.

**Methods:**

Human primary proximal tubule epithelial cells (hPTECs) and clonal tubular epithelial cells (HK2) were treated with Transforming Growth Factor Beta1 (TGF-β1) ± apyrase, or ATPγS for 48 h. For inhibitor studies, cells were co-incubated with Cx43 mimetic Peptide 5, or purinergic receptor antagonists Suramin, A438079 or A804598. Immunoblotting, single-cell force spectroscopy and trans-epithelial electrical resistance assessed protein expression, cell-cell adhesion and paracellular permeability. Carboxyfluorescein uptake and biosensing measured hemichannel activity and real-time ATP release, whilst a heterozygous Cx43^+/−^ mouse model with unilateral ureteral obstruction (UUO) assessed the role of Cx43 in vivo.

**Results:**

Immunohistochemistry of biopsy material from patients with diabetic nephropathy confirmed increased expression of purinergic receptor P2X7. TGF-β1 increased Cx43 mediated hemichannel activity and ATP release in hPTECs and HK2 cells. The cytokine reduced maximum unbinding forces and reduced cell-cell adhesion, which translated to increased paracellular permeability. Changes were reversed when cells were co-incubated with either Peptide 5 or P2-purinoceptor inhibitors. Cx43^+/−^ mice did not exhibit protein changes associated with early tubular injury in a UUO model of fibrosis.

**Conclusion:**

Data suggest that Cx43 mediated ATP release represents an initial trigger in early tubular injury via its actions on the adherens and tight junction complex. Since Cx43 is highly expressed in nephropathy, it represents a novel target for intervention of tubulointerstitial fibrosis in CKD.

**Video Abstract**

**Graphical abstract:**

In proximal tubular epithelial cells (PTECs), tight junction proteins, including zona occuludens-1 (ZO-1), contribute to epithelial integrity, whilst the adherens junction protein epithelial (E)-cadherin (ECAD) maintains cell-cell coupling, facilitating connexin 43 (Cx43) gap junction-mediated intercellular communication (GJIC) and the direct transfer of small molecules and ions between cells. In disease, such as diabetic nephropathy, the pro-fibrotic cytokine transforming growth factor beta1 (TGF-β1) binds to its receptor and recruits SMAD2/3 signalling ahead of changes in gene transcription and up-regulation of Cx43-mediated hemichannels (HC). Uncoupled hemichannels permit the release of adenosine triphosphate (ATP) in to the extracellular space (↑[ATP]_e_), where ATP binds to the P2X7 purinoreceptor and activates the nucleotide-binding domain and leucine-rich repeat containing (NLR) protein-3 (NLRP3) inflammasome. Inflammation results in epithelial-to-mesenchymal transition (EMT), fibrosis and tubular injury. A major consequence is further loss of ECAD and reduced stickiness between cells, which can be functionally measured as a decrease in the maximum unbinding force needed to uncouple two adherent cells (Fmax). Loss of ECAD feeds forward to further lessen cell-cell coupling exacerbating the switch from GJIC to HC-mediated release of ATP. Reduction in ZO-1 impedes tight junction effectiveness and decreases trans-epithelial resistance (↓TER), resulting in increased paracellular permeability. 
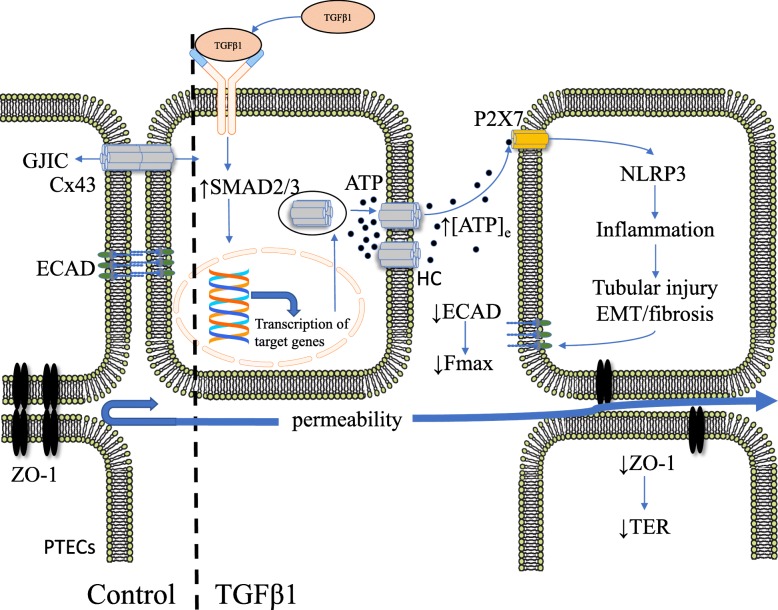

## Background

Affecting 10% of the global population and increasing in prevalence annually, Chronic Kidney Disease (CKD) is a health crisis from which millions die in the absence of a definitive treatment [1]. Individuals are more in danger of CKD if they have any one or more of a number of risk factors including high blood pressure, smoking, established cardiovascular disease and obesity [2]. Characterized by persistent inflammation and fibrosis, tubulointerstitial injury of the proximal region is the key underlying pathology of CKD and develops in response to a number of morphological and phenotypic changes culminating in loss of epithelial stability and increased extracellular matrix deposition [3, 4]. Despite our understanding of these changes, knowledge of the stimuli that initiate activation of resident primary tubular epithelial cells remains limited.

In recent years, trans-membrane proteins called connexins have attracted considerable interest as a potential future target for treatment of multiple disease states [5–8], including various forms of CKD [9]. Connexins assemble into hexameric structures called hemichannels, forming pores in the membrane which directly link the cytoplasm of adjacent cells through the formation of gap junctions [10]. In the absence of cell-cell adhesion, gap junctions fail to form and uncoupled hemichannels allow paracrine release of adenosine triphosphate (ATP) into the local extracellular environment [11].

In CKD, diabetic nephropathy accounts for approximately 50% of patients presenting with end-stage renal failure [1]. Hyperglycemia and downstream changes in connexin expression are critical in the development and progression of secondary micro-vascular complications, [12–15] with glucose decreasing gap junction conductance and disrupting cellular homeostasis in a variety of cell types [16–19]. Evidence that connexin expression is linked to renal damage in CKD [9, 11, 12, 20–24], suggests that they represent a viable therapeutic target for treatment of the disease. Recent studies have confirmed elevated levels of predominant isoform Cx43, in human and rodent models of early renal disease [20, 21] whilst the Cx43^+/−^ unilateral ureteral obstruction (UUO) mouse demonstrates reduced collagen deposition and macrophage infiltration [24]. Work within our group confirmed increased expression of Cx43 in the tubular region of biopsy material isolated from patients with diabetic nephropathy [11]. Despite increased expression, Transforming Growth Factor Beta, the main pro-fibrotic cytokine linked to tubular injury in both diabetic nephropathy and other forms of CKD [25, 26], reduced gap junction mediated intercellular communication (GJIC) and increased hemichannel mediated ATP release [11, 27]. With elevated levels of nucleotides and altered connexin expression linked to inflammation and fibrosis in multiple tissue types [28–31], it seems plausible that the loss of GJIC and the subsequent switch to Cx43 mediated hemichannel ATP release in the proximal tubule may be instrumental in facilitating tubular injury.

Loss of E-cadherin (ECAD) mediated cell-cell adhesion is pivotal in initiating a series of phenotypic and morphological events that precede tubulointerstitial fibrosis [27, 32, 33], with the disassembly of the adherens junction (AJ) and tight junction (TJ) complexes linked to loss of epithelial stability, inflammation, fibrosis and impaired renal function [34, 35]. In the present study, we combine in vivo and in vitro models of CKD to investigate if targeting Cx43 expression and hemichannel activity through genetic and pharmacological blockade, could negate loss of markers linked with tubular injury, ultimately improving function through diminished hemichannel activity, restoration of E-cadherin mediated cell-cell adhesion and reduced paracellular permeability. Using an array of techniques to assess both changes in expression (immunohistochemistry, immunoblotting) and changes in function (e.g. ATP biosensing, single-cell force spectroscopy and trans-epithelial resistance) we demonstrate that increased Cx43 expression and hemichannel mediated ATP release promotes disassembly of adherens and tight junction complexes in human primary proximal tubule epithelial cells and in the UUO mouse model of advanced fibrosis in which the initiating cause is tubulointerstitial inflammation [24, 36]. Blockade of hemichannel mediated ATP release by the Cx43 mimetic Peptide 5, reduces disassembly of the adherens/tight junction complex and restores epithelial integrity. Importantly, these observations were paralleled in the Cx43^+/−^ UUO model, where Cx43 expression is reduced by 50%. Up-regulated in renal tubules of people with diabetic nephropathy (DN) and linked to multiple models of disease and fibrosis [37–40], P2X7 appears instrumental in mediating these ATP driven effects. Together, our study indicates that aberrant Cx43-mediated ATP release may represent a future therapeutic target in preventing early tubular injury linked to tubulointerstitial fibrosis (TIF) in CKD.

## Methods

### Animal model

Three-month-old SV129 Cx43^+/−^ male mice and age-matched wild-type littermates (*n* = 7) were generated. Unilateral ureteral obstruction surgery was performed as describe previously [41]. Non-obstructed kidneys were used as controls. Mice were handled in strict accordance with good animal practice, as defined by the relevant national animal welfare bodies of France, and all work was approved by the appropriate committee of the National Institute for Health and Medical Research (INSERM) and the Sorbonne Université (Paris, France). Animals were housed at constant temperature with ad libitum access to water and food.

### Biopsy staining

Kidney biopsy slices were obtained from patients with biopsy-proven diabetic nephropathy (DN) (mean age 55; mean diabetes duration: 6.2 yrs.; mean HbA1c: 7.2%; mean serum creatinine: 464 μmol/L; mean proteinuria: 5.80 g/24 h, *n* = 10). Control renal tissue was obtained from 5 nephrectomy specimens treated for renal carcinoma (mean age 61; mean serum creatinine: 87.8 μmol/L, *n* = 6). As described previously [42], paraffin-embedded renal sections (4 μm) were de-paraffinized, rehydrated and subjected to microwave-based antigen retrieval in citric buffer solution (10 mM), followed by quenching in 1% H_2_O_2_ and blocking in 2% BSA solution. Sections were stained overnight with anti-P2Y2 (Santa-Cruz, 1:50), anti-P2Y6 (Novus Biologicals, 1:250) or anti-P2X7 (Novus Biologicals, 1:200) antibodies and subsequently incubated with DAKO EnVision^+^ System-HRP antibodies (Dako, Carpinteria, CA). Rabbit IgG was incubated with both DN and control renal sections for isotype-matched negative controls. DAB substrate was used for visualisation and sections were counterstained with hematoxylin. Quantitative analysis was measured by Image-Pro Plus 6.0 and presented as a value of integrated optical density (IOD).

### Cell culture and treatment

Human kidney (HK2) cells (passage 18–30) were maintained in DMEM/Hams F12 medium, supplemented with 10% FCS wt/vol, glutamine (2 mmol/l) and EGF (5 ng/ml), in a humidified atmosphere at 37 °C with 5% CO_2_. Cells are proximal tubular epithelial cells, immortalized by the transduction of human papilloma virus 16 (HPV-16) E6/E7 genes and are mycoplasma-free. For all treatments, cells were seeded in low-glucose DMEM/F12 (5 mmol/L) for 48 h, then serum-starved overnight prior to treatment with Transforming Growth Factor Beta-1 (TGF-β1) (2-10 ng/mL) or ATPγS (1-100 μM) for 48 h. For ATP experiments, cells were incubated with either TGF-β1 (10 ng/mL) or ATPγS (100uM) ± ATP-diphosphohydrolase apyrase (5Units/ml); Suramin (100 μM), A438079 (50 μM) or A804598 (50 nM) for 48 h.

Primary human proximal tubule epithelial cells (hPTECs) were maintained in the basal medium obtained from the American Type Culture Collection (ATCC), supplemented with the renal epithelial cell growth kit (PCS-400-040) in a humidified atmosphere at 37 °C with 5% CO_2_. Cells were treated with TGF-β1 (10 ng/mL) +/− Peptide 5 (25 μM). A scrambled version was used as a control.

### Immunohistochemistry

Immunohistochemical staining of paraffin-embedded sections (3 μm-thick) of mouse renal cortex was performed as described previously [41]. After blocking, sections were incubated with antibodies for N-cadherin (NCAD) (ThermoFischer Scientific, PA5–17526, 1:100) and Zona Occludin-1 (ZO-1) (ThermoFischer Scientific, 61–7300, 1:100), and the Envision detection kit for 30mins at RT (DakoFrance, Trappes, France). Negative controls were obtained by the removal of primary antibodies. The immuno-complex was visualised using DAB substrate and counterstained with hematoxylin.

### Total RNA extraction and quantitative real-time PCR

Total RNA was extracted from renal cortex using TRIzol reagent (Euromedex) RNA quality was checked by control of optical density (OD) at 260 and 280 nm. cDNA was synthesized from 1 mg RNA using the Fermentas H Minus First-Strand cDNA Synthesis Kit according to the manufacturer’s instructions. Quantitative PCR experiments were performed as previously described [24]. Each sample was run in triplicate, and analysis of relative gene expression was done by using the 2 ^−ΔΔCT^ method. Results are expressed in graphs as arbitrary units, which represent the ratio of the target gene to the internal control gene (*HPRT*). Sequences of primers used in our studies are listed in Table 1.

**Table 1:** Forward (FW) and reverse (RV) sequences for primers used in qPCR analysis

Gene	Sequence
P2Y2	FW: TCAAACCGGCTTATGGGACC RV: GGCAGCTGAGGTCAAGTGAT
P2Y6	FW: GGGTAGTGTGTGGAGTCGTG RV: AGCGAGTAGACAGGATGGGT
P2X7	FW: GCACGAATTATGGCACCGTC RV: CCCCACCCTCTGTGACATTC

### Western blotting

Preparation of cytosolic protein from human proximal tubule cells, separation by SDS-gel electrophoresis and transfer onto Immobilon-Fl PVDF membranes have been described previously [43]. Membranes were blocked with Odyssey blocking buffer (LI-COR), then probed with specific polyclonal antibodies against E-cadherin (1:1000), N-cadherin (1:1000), β-catenin (1:2000), Claudin-2 (1:500) and ZO-1 (1:1000). Bands were visualized using an OdysseyFC and semi-quantified using ImageStudio (v5.2, LI-COR). Immunoblotting of protein obtained from mouse cortical tissue was performed as above using: E-cadherin (Santa Cruz Biotechnology, sc-7870, 1:500), β-catenin (BD Transduction Laboratories, 610,154, 1:1000) and Claudin-2 (ThermoFisher Scientific, 32–5600, 1:1000).

### Transepithelial electrical resistance

Human renal tubule cells were seeded (6 × 10^4^ cells/ml) onto Transwell filters (12 mm diameter, pore size 0.4 μM; Corning, NY) and incubated with TGF-β1 (10 ng/mL) +/− Peptide 5 (25 μM) for 48 h. Scrambled Peptide 5 (25 μM) was used as a control. Transepithelial electrical resistance (TER) was calculated in ohms/cm^2^ (Ώ.cm^2^).

### Atomic force microscopy force spectroscopy

Adhesion was characterised using single-cell force spectroscopy (SCFS), as described previously [44, 45]. Tip-less Arrow TL1 cantilevers (Nanoworld AG, Switzerland) with a low spring constant were used (0.03 N/m). Cantilevers were sterilized with UV (10mins), before being functionalised in poly-L-lysine (25 μg/ml, 30mins, RT), and fibronectin (20 μg/ml, 2 h, 37 °C). A single cell was captured at the end of the cantilever with a set force (0.8-1 nN) and contact time (8-10s). After attachment, the cell was left to recover for > 5 min, allowing surface binding. The cantilever-attached cell was brought into contact with a substrate cell, until a 1nN contact force was reached. The two cells were attached for 10s to allow cell-cell adhesion, after which the cantilever was retracted at a constant speed (5 μm/sec). Force-displacement curves were measured until complete detachment (pulling length of 40-90 μm). Each procedure occurred in triplicate with 45 s intervals.

### Carboxyfluorescein

HK2 and hPTEC cells were incubated with TGF-β1 (10 ng/mL) ± Peptide 5 (25 μM) or Scrambled (25 μM) for 48 h. Cells were exposed to Ca^2+^-free Balanced Salt Solution (BSS) + carboxyfluorescein (200 μM) for 10 min to permit hemichannel-mediated dye uptake, before reapplying [Ca^2+^]_e_ to close the channels. Images were acquired with a Cool Snap HQ CCD camera (Roper Scientific) and Metamorph software (Universal Imaging Corp., Marlow, Bucks, UK). ImageJ was used to quantify dye uptake, where a region of interest was drawn around each cell (10–15 cells/dish) and mean pixel intensity measured.

### ATP biosensing

ATP-biosensors (Sarissa Biomedical, Coventry UK) were used in simultaneous dual-recording ampomeric mode as described previously [11]. A null sensor accounted for non-specific electro-active artefacts and was subtracted from the ATP trace. Glycerol (2 mM) was included in all solutions. HK2 cells were incubated with TGF-β1 (10 ng/mL) +/− Peptide 5 (25 μM) for 48 h. Cells were transferred to a chamber containing Ca^2+^-containing BSS perfused at 6 ml/min (37 °C) and left for 10 min to acclimatize. Ca^2+^-free BSS was used to open hemichannels and Ca^2+^-containing BSS to close them. Data was calibrated using 10 μM ATP. Recordings were acquired at 4 Hz with a Micro CED (Mark2) interface using Spike software.

### Analysis

Statistical analysis was performed via one-way ANOVA test with Tukey’s multiple comparison post-test or t-test. Data are expressed as mean ± SEM, with ‘n’ denoting sample number. A *p*-value < 0.05 denotes statistical significance.

## Results

### TGF-β1 regulates expression of adherens and tight junction proteins

**Fig. 1:**
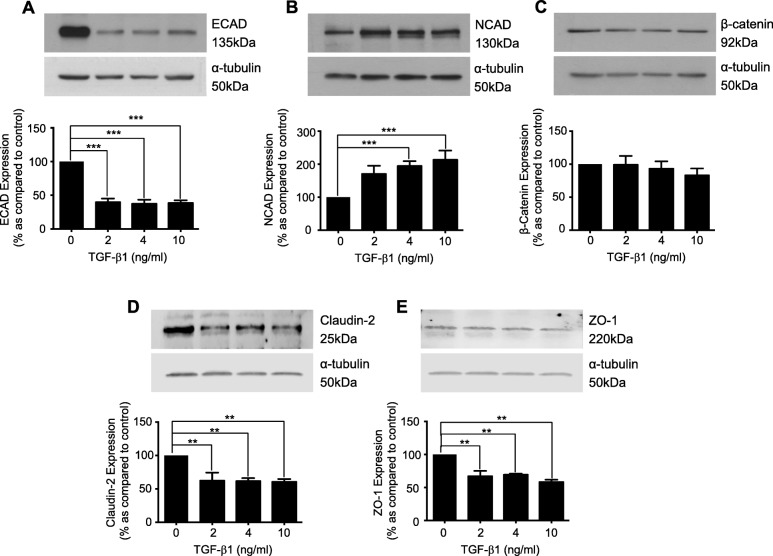
TGF-β1 regulates the expression of adherens and tight junction proteins in cultured human renal tubule cells. Cells were cultured in low glucose control (0) ± TGF-β1 (2-10 ng/mL) for 48 h. Whole-cell expression of E-cadherin (**a**), N-cadherin (**b**), β-catenin (**c**), Claudin-2 (**d**), and ZO-1 (**e**) was confirmed via immunoblotting. Representative blots for each protein are shown, with expression normalized by re-probing for ɑ-tubulin as a loading control. Bars correspond to their associated lanes in the respective blot. Results were from three or more separate experiments; with key significance shown: **P < 0.01; ***P < 0.001

Human Kidney proximal tubule epithelial cells were treated with TGF-β1 (2-10 ng/mL) for 48 h. TGF-β1 downregulated E-cadherin (ECAD) to 39.7 ± 5.5%, 37.3 ± 6.2% and 38.5 ± 4.1% as compared to control, and upregulated N-cadherin (NCAD) to 170.5 ± 24.7%, 194.2 ± 15% and 213.3 ± 28% at 2, 4 and 10 ng/mL TGF-β1 respectively (Fig. 1a & b). TGF-β1 did not alter β-catenin expression (Fig. 1c). Expression of the tight junction protein Claudin-2, was reduced to 62.3 ± 12.2%, 61.6 ± 4.5% and 60.5 ± 4.4% of control at 2, 4 and 10 ng/mL TGF-β1 (Fig. 1d), whilst ZO-1 decreased to 67.0 ± 8%, 69.2 ± 1.7% and 64.8 ± 4.1% respectively (Fig. 1e).

### ATPγS regulates adherens and tight junction proteins

**Fig. 2:**
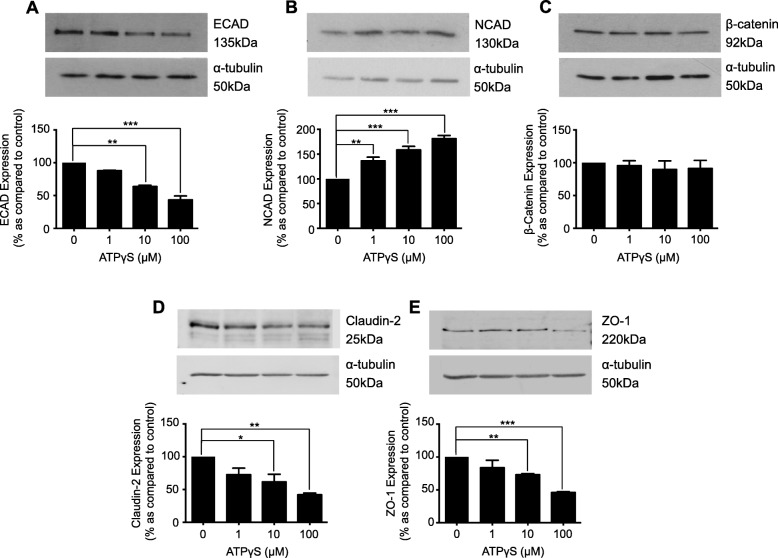
ATPγS regulates the expression of major adherens and tight junction proteins in cultured human renal tubule cells. Cells were cultured in low glucose control (0) ± ATPγS (1-100 μM) for 48 h. Whole-cell expression of E-cadherin (**a**), N-cadherin (**b**), β-catenin (**c**), Claudin-2 (**d**) and ZO-1 (**e**) were assessed through immunoblotting. Representative blots for each protein are shown, with expression normalized by re-probing for ɑ-tubulin as a loading control. Bars correspond to their associated lanes in the respective blot. Results were from three or more separate experiments; with key significance shown: *P < 0.05; **P < 0.01; ***P < 0.001

We previously demonstrated that TGF-β1 increased hemichannel-mediated ATP release [11]. To determine if ATP mediates changes in adherens and tight junction protein expression, human kidney proximal tubule cells were incubated with ATPγS (1-100 μM) for 48 h. The non-hydrolysable P2-agonist decreased ECAD expression to 87.8 ± 0.9%, 63.8 ± 1.9% and 43.4 ± 6.1% as compared to control at 1, 10 and 100 μM ATPγS, and increased NCAD expression to 136.2 ± 7.6%, 158.4 ± 7.3% and 181.3 ± 6.3% respectively (Fig. 2a & b). No change in β-catenin expression was observed (Fig. 2c). ATPγS decreased Claudin-2 to 72.7 ± 9.8%, 61.6 ± 11.6% and 42 ± 2.6% of control (Fig. 2d) and ZO-1 to 83.7 ± 11.4%, 73 ± 1.8% and 45.9 ± 1.4% (Fig. 2e) at 1, 10 and 100 μM respectively.

### ATP is downstream of TGF-β1 in regulating expression of adherens junction proteins

**Fig. 3:**
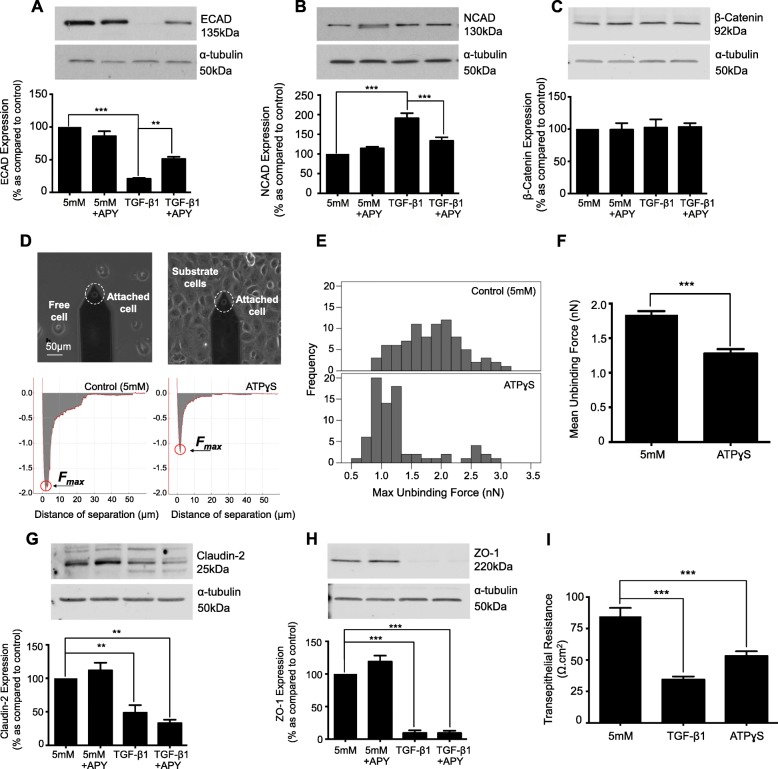
The ATP-diphosphohydrolase, apyrase negates TGF-β1-evoked changes in expression of the adherens junction complex, but fails to restore TGF-β1 induced changes in tight junction protein expression. To determine whether ATP mediates TGF-β1-evoked changes in the expression of adherens and tight junction proteins, cells were cultured in low glucose (5mM) ± TGF-β1 (10 ng/mL) ± apyrase (5 U/ml) for 48 h. Whole-cell expression of E-cadherin (**a**), N-cadherin (**b**), β-catenin (**c**), Claudin-2 (**g**), and ZO-1 (**h**) were assessed via immunoblotting. Representative blots for each protein are shown, with expression normalized by re-probing for ɑ-tubulin as a loading control. Bars correspond to their associated lanes in the respective blot. Changes in expression were matched to changes in function (**d**-**f**) and (**i**). Atomic Force Microscopy single-cell force spectroscopy was used to measure the maximum unbinding force required to uncouple two adhered cells. Human renal tubule cells were cultured in low glucose with/without ATPγS (100 μM) for 48 h. Retraction Force-displacement curves for control and ATPγS (**d**) cells are shown respectively. Maximum unbinding forces (**e** & **f**) between two cells was determined by measuring the amplitude of the points circled in red (**d**). Lastly, disassembly of the tight junction complex is paralleled by loss of transepithelial electrical resistance (TER). Cells were cultured in low glucose ± either TGF-β1 (10 ng/mL) or ATPγS (100 μM) for 48 h on Transwell inserts. Data is expressed as mean ± SEM of multiple cells from 4 separate experiments, with key significance shown: **P < 0.01, ***P < 0.001

To delineate a downstream role for ATP, human proximal renal tubule cells were incubated with TGF-β1 (10 ng/mL) ± apyrase (5 U/ml) for 48 h. As expected, TGF-β1 (10 ng/mL) decreased ECAD expression to 20.9 ± 1.4%, whilst co-incubation with apyrase partially restored expression (51.2 ± 3.2%, Fig. 3a). Apyrase reduced the TGF-β1 evoked increase in NCAD from 191.1 ± 12.6% to 133.3 ± 9.1%, as compared to control (Fig. 3b). TGF-β1 failed to significantly change β-catenin expression (Fig. 3c).

Our previous work confirmed that TGF-β1 reduces E-cadherin mediated adhesion in human renal tubule cells [11]. On this basis, we assessed the effect of ATP on cell-to-cell tethering. A single cell (±100 μM ATPγS, 48 h) was attached to a tip-less cantilever (probe cell) and brought into contact with a substrate cell (Fig. 3d). The two cells were attached with fixed force (1nN) and contact time (10s) after which the probe cell was retracted. Force versus displacement was recorded continuously. The zero axis represents the baseline in which the two cells were completely detached, whilst the lowest negative point below the baseline represents maximum unbinding force (F_max_). Analysis determined a reduction in the mean unbinding forces between cells treated with ATPγS (Fig. 3d & f) from 1.836nN +/− 0.055 to 1.279nN +/− 0.064. Furthermore, a reduction in force variability occurs (Fig. 3e).

Apyrase failed to negate TGF-β1-evoked changes in tight junction protein expression. As expected, TGF-β1 (10 ng/mL) reduced expression of Claudin-2 (Fig. 3g) and ZO-1 (Fig. 3h) to 48.6 ± 11.3% and 9.5 ± 4% respectively, compared to the control. Co-incubation with apyrase failed to negate this reduction, with expression remaining at 33.3 ± 4.9% and 9.5 ± 3.5% for Claudin-2 and ZO-1 respectively. Trans-epithelial electrical resistance (TER) confirmed loss of tight junction function in cells cultured with TGF-β1 (10 ng/mL) or ATPγS (100 μM) for 48 h. TER confirmed that TGF-β1 and ATPγS decreased epithelial resistance from 67.7 ± 5.5 Ω.cm^2^ to 27.6 ± 2 Ω.cm^2^ and 42.6 ± 3 Ω.cm^2^ respectively (Fig. 3i).

### Purinoreceptor, P2X7, exhibits increased expression in our in vitro and in vivo models of disease

**Fig. 4:**
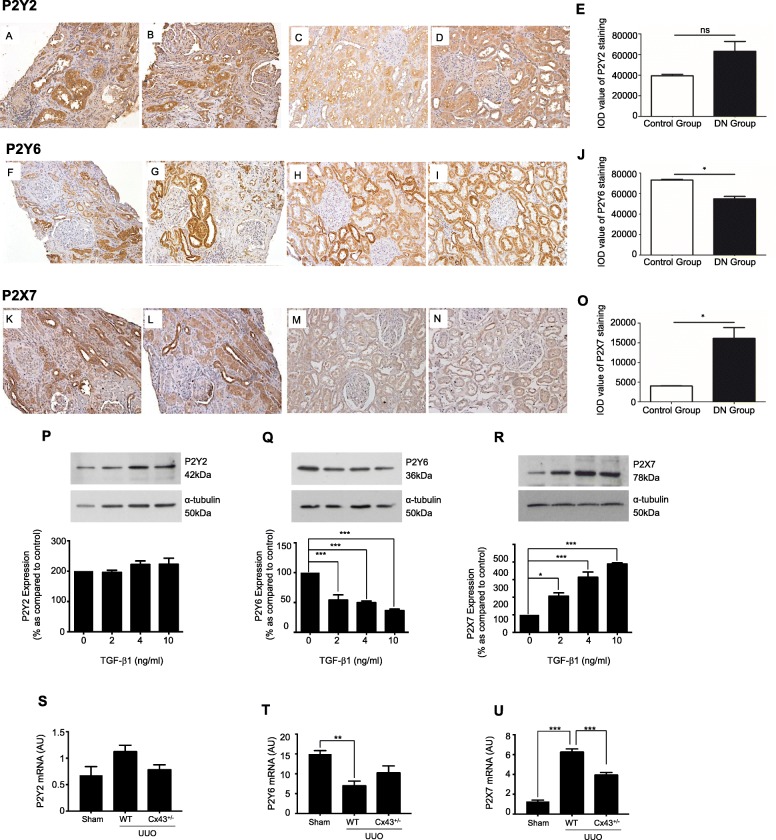
P2X7 is upregulated in renal tubule cells exposed to TGF-β1 and in patients with CKD and in the UUO mouse mdoel. Staining of biopsy material isolated from individuals with DN was performed for P2Y2 (**a** & **b**), P2Y6 (**f** & **g**), and P2X7 (**k** & **l**). Staining for non-diabetic controls can be observed in P2Y2 (**c** & **d**), P2Y6 (**h** & **i**), and P2X7 (**m** & **n**). Magnification: 400X. hPTECs were also cultured in low glucose with/without TGF-β1 (2-10 ng/mL) for 48 h. Whole-cell abundance of P2Y2 (**p**), P2Y6 (**q**) and P2X7 (**r**) were determined through densitometry. Representative blots for each protein are shown, with expression normalized by re-probing for ɑ-tubulin as a loading control. Bars correspond to their associated lanes in the respective blot. qRT-PCR was performed on tubules isolated from WT (UUO) and Cx43^+/−^ (UUO) for P2Y2 (**s**), P2Y6 (**t**) and P2X7 (U). qRT-PCR are expressed in graphs as arbitrary units (a.u.) that represent the ratio of the target gene to internal control gene (*HPRT*). Results were from three or more separate experiments; with key significance shown: *P < 0.05; ***P < 0.001

Immunohistochemistry of purinoreceptor isoforms P2Y2, P2Y6 and P2X7 (Fig. 4) in biopsy material isolated from people with and without DN confirmed upregulation of P2Y2 (Fig. 4a & b) (IOD: 63,120 ± 9485 versus 39,400 ± 142 for control, Fig. 4c & d), with a decrease in P2Y6 (Fig. 4f & g) (IOD: 54,850 ± 2337 versus 73,170 ± 574 for control, Fig. 4h & i). P2X7 expression was significantly increased (Fig. 4k & l) (IOD: 16,130 ± 2715 compared to 4013 ± 62, for control; Fig. 4m & n).

To confirm if TGF-β1 regulates purinoreceptor expression in vitro, cells were incubated with TGF-β1 (2-10 ng/mL) for 48 h. TGF-β1 failed to significantly change P2Y2 expression (Fig. 4p) yet a significant down-regulation of P2Y6 to 54.1 ± 8.8%, 49.8 ± 2.8% and 36.6 ± 2.5% at 2, 4 and 10 ng/mL TGF-β1 respectively was observed (Fig. 4q). In contrast, a significant increase in P2X7 was observed at 2, 4 and 10 ng/mL TGF-β1 (206.4 ± 18.7%, 313.3 ± 29.9% and 389.1 ± 6.2% respectively, Fig. 4r).

Lastly, quantitative PCR experiments confirmed that purinergic receptor expression in tubules isolated from the UUO mouse model exhibit a similar pattern of expression to that observed in human biopsy material and in our TGF-β1 treated cells. qRT-PCR of purinoreceptor isoforms P2Y2, P2Y6 and P2X7 (Fig. 4s-u) confirmed upregulation of P2Y2 (Fig. 4s) (0.68 ± 0.16 to 1.1+/− 0.11), with a decrease in P2Y6 (Fig. 4t) (15.0 ± 0.86 to 7.2 ± 0.97) and a significant increase in P2X7 (Fig. 4u) expression (1.3 ± 0.15 to 6.3 ± 0.25) as compared to WT control.

### Blocking P2X7 negates TGF-β1-evoked changes in adherens & tight junction proteins

Purinergic receptor P2X7, has been linked to fibrosis in multiple disease states [37–40]. Given the statistically significant increase in P2X7 expression in both human biopsy material and in our UUO mouse, the pathophysiological role of P2X7 in mediating the downstream effects of ATP induced tubular injury in our model system were investigated.

**Fig. 5:**
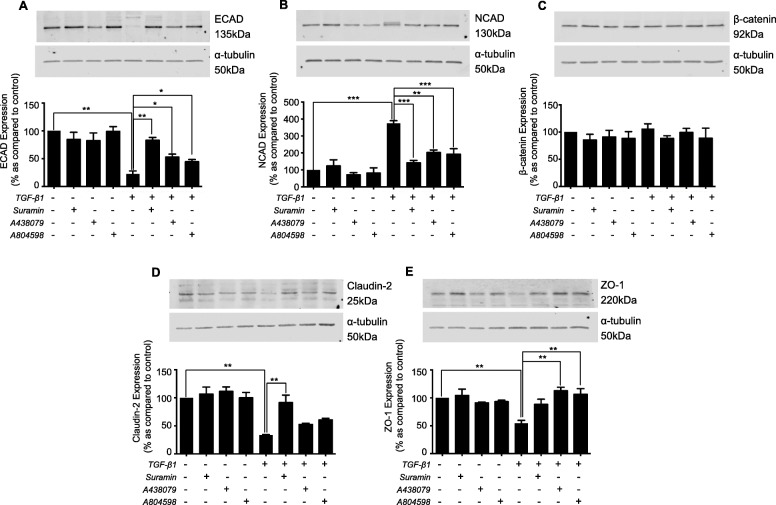
Blockade of purinoreceptors attenuates TGF-β1 induced changes in expression of adherens and tight junction protein. HK2 cells were cultured in low glucose (5mM) ± TGF-β1 (10 ng/mL) ± either: Suramin (100 μM), A438079 (50 μM) or A804598 (50 nM) for 48 h. Whole-cell expression of E-cadherin (**a**), N-cadherin (**b**), β-catenin (**c**), Claudin-2 (**d**) and ZO-1 (**e**) were assessed via immunoblotting. Representative blots for each protein are shown, with expression normalized by re-probing for ɑ-tubulin as a loading control. Bars correspond to their associated lanes in the respective blot. Results were from three or more separate experiments; with key significance shown: *P < 0.05; **P < 0.01; ***P < 0.001

To determine if TGF-β1 evoked changes in hemichannel mediated ATP release, facilitates its effects via activation of P2X7, HK2 cells were treated with TGF-β1 ± Suramin (100 μM) or P2X7-specific inhibitors A438079 (50 μM) or A804598 (50 nM). The TGF-β1-evoked loss in ECAD expression (Fig. 5a), was partly restored, from 22.2 ± 5.5% to 83.1 ± 5% (Suramin), 52.8 ± 5.4% (A438079) and 44.4 ± 4% (A804598), whilst NCAD expression (Fig. 5b) decreased from 374 ± 16.3% to 141.8 ± 14.4% (Suramin), 202.5 ± 15% (A438079) and 192.5 ± 32.6% (A804598). No change in β-catenin was observed (Fig. 5c).

Inhibition of P2X7 partially reversed loss of tight junction protein expression evoked by TGF-β1. Claudin-2 (Fig. 5d) levels were restored from 33.4 ± 1% to 91.5 ± 13.4% (Suramin), 52.6 ± 2.1% (A438079) and 60.7 ± 2.8% (A804598) and ZO-1 (Fig. 5e) returned from 54.5 ± 5.2% to 88.3 ± 9.3% (Suramin), 112.8 ± 6.3% (A438079) and 106.4 ± 10.1% (A804598). For all experiments, inhibitors alone failed to induce any significant change in expression.

### Peptide 5 inhibits TGF-β1-induced Cx43 hemichannel activity and ATP release

**Fig. 6:**
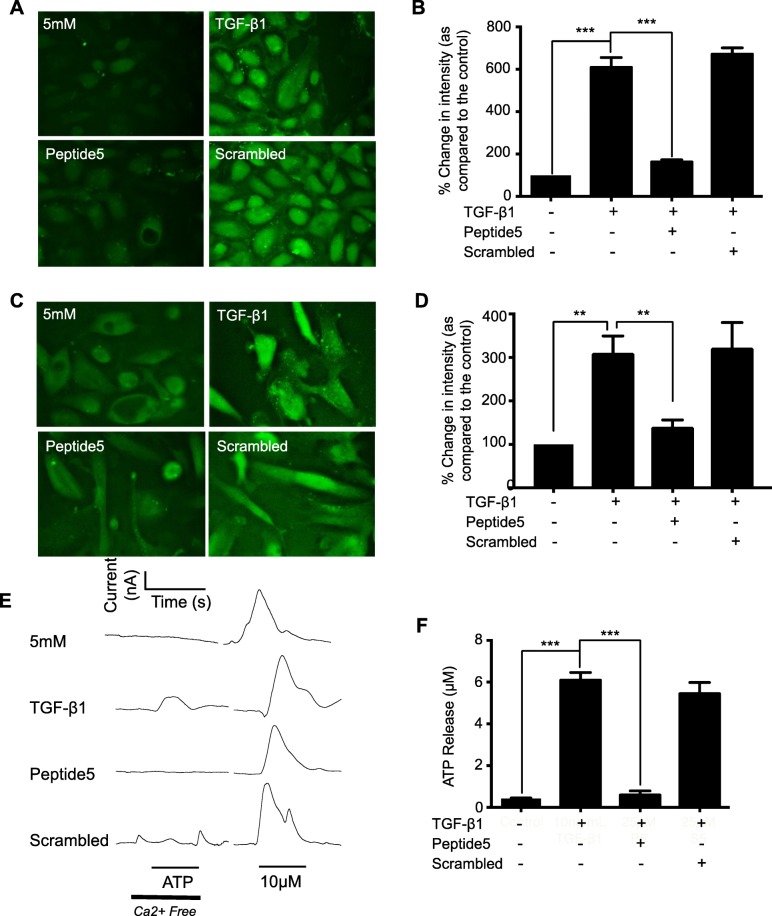
Co-incubation of TGF-β1 treated renal tubule cells with Cx43 mimetic, Peptide 5, impairs hemichannel activity and ATP release. HK2 cells and hPTECs were cultured on either fluorodishes or glass coverslips in low-glucose (5mM) ± TGF-β1 (10 ng/mL) ± Peptide 5 (25 μM) for 48 h. A carboxyfluorescein uptake assay (**a** & **c**) assessed hemi-channel activity in HK2 and hPTEC cells respectively. Minimal dye uptake was observed in control cells, whilst strong dye loading occurred in TGF-β1 treated cells. Co-incubation with Peptide 5 attenuated dye loading, whilst a scrambled control had no significant effect. Pixel intensity of dye loading was quantified and compared to the low-glucose control (**b** & **d**) for 10 cells in 3 separate experiments. Biosensors were used to measure hemi-channel dependent release of ATP. Representative traces (−null) are shown (**e**). The amplitude of any major ATP peak was measured and compared to control recordings, where little response occurred in response to hemi-channel opening following the removal of calcium. TGF-β1-treated cells exhibit significant ATP in response to hemi-channel opening, an effect negated when co-incubated with Peptide 5 (25 μM) (**f**). Exogenous ATP was administered at the end of each experiment ensuring effective calibration. Data is expressed as mean ± SEM of multiple cells from 4 separate experiments, with key significances shown: **P < 0.01, ***P < 0.001

To study the efficacy of Peptide 5 in blocking Cx43 mediated hemichannel activity in renal tubule epithelial cells, HK2 and hPTECs were incubated in TGF-β1 (10 ng/mL) ± Peptide 5 (25 μM) for 48 h. Dye uptake increased in TGF-β1-treated HK2 cells to 609.4 ± 46% compared to unstimulated control (Fig. 6a & b), whilst co-incubation with Peptide 5 reduced uptake to 163 ± 10.2%. Co-incubation with scrambled peptide failed to blunt the TGF-β1 response (671.5 ± 29.5%, Fig. 6a & b). The effects were matched in primary hPTECS, where Peptide 5 significantly reduced uptake to 141.7 ± 16.3% from 311.2 ± 39.6% in TGF-β1 treated cells (Fig. 6c & d). Scrambled control failed to reverse the TGF-β1 effects (323.3 ± 58.1%).

Biosensing uses enzymatically-coated electrodes to measure real time ATP release. Cultured as above, ATP release increased from 0.43 ± 0.03 μM to 6.10 ± 0.36 μM in TGF-β1-treated cells (Fig. 6e & f). Peptide 5 successfully prevented ATP release, restoring levels to 0.60 ± 0.20 μM. Scrambled peptide in the presence of TGF-β1 had no affect (5.45 ± 0.53 μM, Fig. 6e & f).

### Peptide 5 negates TGF-β1 induced disassembly of the adherens and tight junction complex in human primary renal tubule cells

**Fig. 7:**
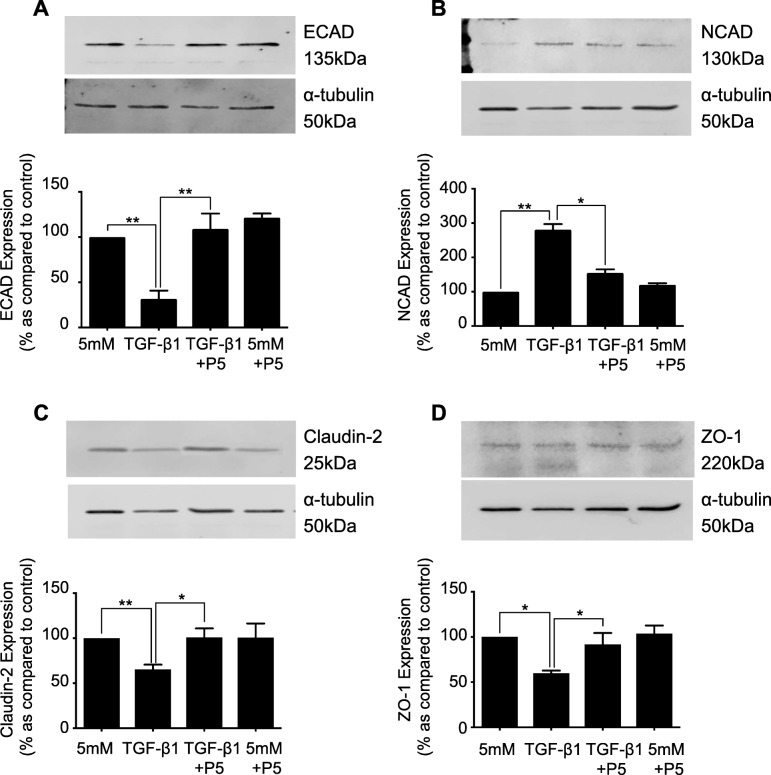
Blockade of Cx43-mediated hemichannel ATP release, negates the TGF-β1-induced disassembly of the tight and adherens junction complex. hPTECs were cultured in low glucose (5mM) ± TGF-β1 (10 ng/mL) ± Peptide 5 (25 μM) for 48 h. Expression of E-cadherin (**a**), N-cadherin (**b**), Claudin-2 (**c**) and ZO-1 (**d**) were assessed via western blotting. Representative blots for each protein are shown, with expression normalized by re-probing for ɑ-tubulin as a loading control. Bars correspond to their associated lanes in the respective blot. Results were from three or more separate experiments; with significance shown: *P < 0.05; **P < 0.01

Having confirmed the efficacy of Peptide 5, a role for TGF-β1 induced Cx43 mediated hemichannel ATP release in initiating early changes of tubular injury were assessed. Human renal proximal tubule epithelial cells were incubated with TGF-β1 (10 ng/mL) ± Peptide 5 (25 μM) for 48 h. Co-incubation of hPTECs with TGF-β1 and Peptide 5 restored ECAD expression (Fig. 7a) from 31.5 ± 9.2% to 108.9 ± 17.1%; NCAD expression (Fig. 7b) from 280.5 ± 16.7% to 154.7 ± 10.6%; Claudin-2 expression (Fig. 7c) 65.3 ± 5.4% to 100.9 ± 10% and finally ZO-1 expression (Fig. 7c) from 59.63 ± 3.1% to 91.6 ± 12.8% as compared to low-glucose control. Incubation of cells with either Peptide 5 alone or a scrambled version of Peptide 5, failed to change expression of our candidate proteins.

### Regulation of junctional components in UUO mice is Cx43-dependent

**Fig. 8:**
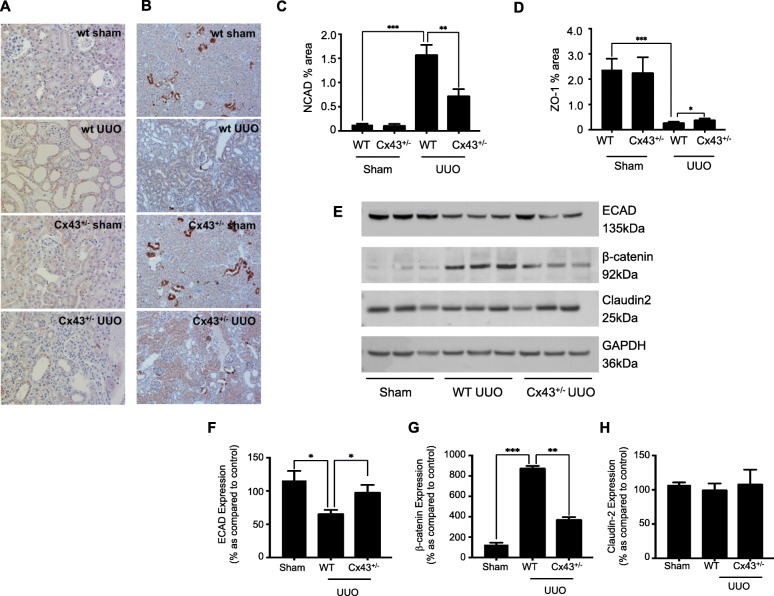
Cx43^+/−^ mice exhibit minimal disassembly of the adherens and tight junction complex. Quantification of immunohistochemical staining determined an increase in N-cadherin (**a** & **c**) and decrease in ZO-1 (**b** & **d**) expression in wildtype (WT) UUO compared to WT sham controls. In Cx43^+/−^ (UUO) mice, expression of both markers was partially restored to near basal levels. Furthermore, western blotting (**e**) of renal cortex determined changes in expression of E-cadherin (**f**), β-catenin (**g**) and Claudin-2 (**h**) in WT UUO mice compared to WT shams, congruent to those found in vitro when cells were treated with TGF-β1. As expected, these changes were partially negated in Cx43^+/−^ (UUO) mice. Results were from six separate experiments; with significance shown: *P < 0.05; **P < 0.01; ***P < 0.001

Having confirmed a role for Cx43 mediated ATP release in TGF-β1 induced tubular injury pharmacologically, the expression of key candidate proteins was assessed in vivo using our Cx43^+/−^ mouse model of UUO. Quantification of immunohistochemistry determined an increase in N-cadherin from 0.131 ± 0.02% to 1.59 ± 0.19% (Fig. 8a & c) and a decrease in ZO-1 from 2.38 ± 0.43% to 0.293 ± 0.02% (Fig. 8b & d) in UUO mice compared to WT healthy animals. Genetic depletion of Cx43 partially negated these changes, returning expression to 0.734 ± 0.13% (Fig. 8a) and 0.402 ± 0.04% (Fig. 8b) for N-cadherin and ZO-1 respectively. Staining in Cx43^+/−^ sham animals was unaltered from WT sham controls for both proteins.

In accordance with previous data, expression of E-cadherin was downregulated from 115.79 ± 14.34% to 66.61 ± 4.91% (Fig. 8e & f); β-catenin upregulated from 126.04 ± 18.38% to 880.42 ± 16.76% (Fig. 8e & g) and Claudin-2 downregulated from 107.15 ± 3.68% to 100.61 ± 8.63% (Fig. 8e & h), in WT UUO mice as compared to WT shams. In Cx43^+/−^ UUO mice, expression of E-cadherin was restored to 98.73 ± 10.25% (Fig. 8e & f) and β-catenin to 376.43 ± 18.56% (Fig. 8e & g). Claudin-2 exhibited minimal change at 109.08 ± 20.23% (Fig. 8e & h).

## Discussion

Tubulointerstitial fibrosis is the final common pathway in CKD, yet treatment represents an unmet clinical need. The emerging field of connexins suggests that these membrane bound proteins may offer a viable therapeutic target in future treatment of disease [46], with recent studies confirming that increased expression of Cx43 in the proximal region of the diabetic kidney, is accompanied by loss of gap junction mediated intercellular communication and increased hemichannel mediated ATP release [11]. Elevated levels of ATP have been linked to inflammation and fibrosis in multiple disease states [28–31]. And work within our laboratory confirms increased expression of inflammatory and profibrotic markers in ATP treated human primary proximal tubule cells [11]. In the current study we investigate a role for aberrant Cx43 mediated hemichannel activity in mediating the phenotypic and functional changes of early tubular injury [11].

As the key underlying pathology of End Stage Renal Disease, tubulointerstitial fibrosis is partly contributed to, by Epithelial to Mesenchymal Transition (EMT) [32]. EMT occurs in the face of injury as cells attempt to evade apoptosis. In doing so, cells downregulate markers commonly associated with an epithelial phenotype, e.g. E-cadherin, ZO-1, and Claudin-2 whilst upregulating those more commonly associated with a mesenchymal phenotype and increased fibrosis, e.g. α-SMA, N-cadherin, and Snail [32–34]. Initiation is associated with disassembly and breakdown of adherens junctions and tight junctions, culminating in loss of cell adhesion and increased paracellular permeability.

Previous findings from our lab, confirm that TGF-β1 mediates morphological and phenotypic changes characteristic of EMT in both HK2 and hPTECs [11, 27]. Furthermore, TGF-β1 evokes an increase in Cx43 expression, an effect dependent upon SMAD2/SMAD3 signalling [11], and corroborated by multiple studies confirming direct binding of Smad3 and Smad4, to the promoter of *Gja1* [47, 48]. As previously reported in podocytes, it is plausible that TGF-β1 may, via crosstalk with the STAT1 signalling pathway [21] mediate Cx43 hemichannel expression via AKT/p38 signaling and the binding of STAT1/c-Jun to the Cx43 promoter [21]. In the current study; we present novel evidence that TGF-β1 evokes increased Cx43 hemichannel-mediated ATP release, which in turn, contributes to purinergic mediated disassembly of tight junctions and adherens junctions in the proximal region of the diseased kidney.

Our In vitro studies confirm that incubation of renal proximal tubule cells with TGF-β1, or non-hydrolysable ATPγS decreased expression of E-cadherin, Claudin-2 and ZO-1, with increased expression of N-cadherin. To delineate the functional consequences of these altered levels of expression, atomic force microscopy force spectroscopy and trans-epithelial electrical resistance assessed changes in cell-cell tethering and paracellular permeability respectively. Corroborating recent findings that depletion of Claudin-2 and ZO-1 is detrimental to proximal tubule epithelial cell function through a “leaky” epithelia, [49] both TGF-β1 and ATPγS independently reduce PTEC resistance, and ultimately impair barrier integrity. Furthermore, force spectroscopy confirmed ATPγS reduced the unbinding force required to uncouple two attached cells. Co-incubation of TGF-β1 with the ectonucleotidase apyrase, partially restored expression of E-cadherin and N-cadherin, yet failed to negate TGF-β1 evoked changes in tight junction protein expression. These observations, can most likely be explained by studies confirming a role for ATP metabolites in regulating expression of tight junction proteins, [50, 51] and are further supported by observations that TGF-β1 evoked changes in tight junction expression are blunted when cells are co-incubated with adenosine receptor antagonist; Suramin. The origin of this deleterious signal was confirmed in TGF-β1 treated cells preincubated with Peptide 5. Peptide 5 is a 12 amino acid peptide which targets the 2nd extracellular loop of Cx43 [52], it has been proven successful in blocking Cx43 hemichannels when delivered topically, intraocularly [53], into cerbebrospinal fluid and systemically [52]. Multiple approaches have been used to confirm target applicability and specificity and all have yielded similar and significant benefits across different injury models. The ability of Peptide 5 to block hemichannel activity, ATP-release and ultimately disassembly of the adherens/tight junction complex in our model system, was assessed by carboxyfluorescein dye uptake, ATP-biosensing and western blotting respectively. Co-incubation of both HK2 and hPTECS with TGF-β1 and Peptide 5 significantly reduced dye uptake and restored ATP release to near basal, whilst Peptide 5 successfully prevented TGF-β1-evoked changes in expression of E-cadherin, N-cadherin, Claudin-2 and ZO-1 in human primary renal proximal tubule cells.

Confirmation for Cx43 mediated ATP release in initiating these changes of early tubular injury, was confirmed in a Cx43^+/−^ heterogeneous knockout mouse model having undergone unilateral ureteral obstruction (UUO). The UUO model recapitulates the fundamental pathogenic mechanisms that typify all forms of CKD including diabetic nephropathy and is widely used for the study of renal fibrosis and inflammation [36]. Staining renal cortex slices from wildtype UUO mice confirmed a decrease in N-cadherin and ZO-1 expression, whilst immunoblotting of isolated renal cortex protein demonstrated reduced E-cadherin expression, with increased expression of β-catenin. Restoration of expression of the adherens junction proteins in the Cx43^+/−^ UUO model supports our earlier findings where Cx43 block diminished hemichannel mediated ATP release. However, whilst changes in expression of our adherens junction proteins were recapitulated in vivo, minimal change was observed for tight junction protein Claudin-2. Despite this, ZO-1 expression was decreased in vitro and in vivo, an effect restored when hPTEC cells were co-incubated with Peptide 5, or Cx43 activity was suppressed (Cx43^+/−^). The importance of this to tubular function and subsequent injury, is supported by observations, which suggest that ZO-1 is a critical regulator of tight junction assembly [54, 55], with loss of expression linked to defects in tight junction assembly and a severely disrupted paracellular barrier [54–56]. Although not present in the mature adherens junction complex, tight junction biogenesis is characterized by an interaction between ZO-1 and cadherins, an association which allows for fusion of belt-like tight junctions and gap junction formation [57]. In addition, in its capacity as a scaffolding protein, ZO-1 directly facilitates Cx43 mediated gap junction formation [58]. Thus, restoration of ZO-1 expression, through blockade of Cx43 hemichannel mediated ATP release, has potential to not just restore barrier function, but to maintain cell-to-cell adhesion through efficient adherens junction formation, blockade of EMT and restoration of direct GJIC.

With an imbalance in ATP signalling &/or degradation linked to the underlying pathology of multiple diseases [59–61], we investigated a role for downstream purinergic signalling in driving these Cx43 hemichannel mediated events. ATP signals via activation of membrane-bound purinoreceptors [62]. Increased activation of purinergic receptors, notably P2X7, has been linked to inflammatory damage in the renal vasculature, glomerulus and tubular regions in multiple experimental models of kidney disease [62–64]. In diabetic nephropathy, P2X7 expression is associated with mesangial expansion and impaired glomerular filtration [38], whilst genetic and pharmacological (AZ11657312) ablation of P2X7 in a mouse model of Type I diabetes impaired glomerular macrophage infiltration and decreased collagen IV deposition [38]. In human biopsy material from people with diabetic nephropathy and in tubules isolated from the UUO mouse model, we observed a significant upregulation of P2X7 expression, an effect which was blunted when Cx43 expression was genetically reduced (Cx43^+/−^/UUO). Peptide 5, has been shown to negate inflammation and associated tissue damage in multiple systems of disease, specifically those where initial pathology appears perpetuated by activation of the nucleotide-binding domain and leucine-rich repeat containing (NLR) protein-3 (NLRP3) inflammasome [65]. Upregulated in classic immune cells (e.g. infiltrating macrophages) and tubular epithelial cells of the kidney, the NLRP3 inflammasome is a protein complex involved in initiating the innate immune response [66], with activation linked to a variety of glomerular and tubulointerstitial diseases [67, 68]. Although pharmacological inhibition negates inflammation and improves overall tissue function in a host of inflammatory conditions, lack of understanding of structure and underlying regulatory mechanisms of NLRP3 has hindered the discovery and development of successful therapeutics [66].

Stimulated by age-related Damage-associated molecular patterns (DAMPs), including excess ATP, P2X7 mediated activation of the NLRP3 complex triggers a cascade of events that culminate in secretion of downstream pro-inflammatory mediators, including interleukin-1β (IL-1β) and interleukin-18 (IL-18) [66]. A recent study in acute renal injury confirmed that the Cx43^+/−^ mouse exhibits reduced renal NLRP3 expression and decreased serum levels of IL-1β as compared to its wildtype control [69]. Moreover, NLRP3 activation has recently been linked to induction of EMTand diminished ECAD expression in multiple cell types [70, 71], whilst Cx43 mediated NLRP3 activation initiates breakdown and disruption of the retinal pigment epithelium (RPE) at the back of the diabetic eye [65]. In addition, with priming of the NLRP3 inflammasome linked to nuclear factor-kappa B (NF-κB) activation, combined with reports that NF-κB activation binding to the Cx43 gene promoter directly increases Cx43 expression [72], it is possible that inflammasome pathway priming parallels increased Cx43 expression, further perpetuating aberrant Cx43 mediated ATP release [65]. The current study confirms that specific inhibition of P2X7 in PTECS co-incubated with TGF-β1 +/− P2X7 inhibitor A438079 or A804598, negates TGF-β1-evoked changes in expression of EMT markers, E-cadherin, N-cadherin, Claudin-2 and ZO-1. In addition, using AFM-SCFS, we have recently shown that the ATPγS induced downregulation of E-cadherin expression in proximal kidney cells, is paralleled by a P2X7 mediated reduction in intercellular ligation forces, decreased tether rupture events and cytoskeletal remodeling [73]. Combined with observations that Peptide 5 negates TGF-β1 induced disassembly of the adherens junction and tight junction complex, our findings suggest that TGF-β1 evoked changes in Cx43 mediated ATP likely initiates P2X7 mediated tubular injury and EMT via an NLRP3 dependent mechanism.

However, although blockade of P2X7 and an impaired ATP driven response clearly supports a role for purinergic mediated signalling in early tubular injury, recent clinical trials have failed to demonstrate a beneficial effect of P2X7 antagonism in numerous inflammatory illnesses, an effect most likely linked to the genetic variation within the P2X7 [74]. With aberrant Cx43 activity linked to activation of P2X7 and NLRP3, targeting aberrant Cx43 mediated hemichannel ATP release clearly represents a future therapeutic avenue for the treatment of chronic kidney disease and other conditions where inflammation appears to be the underlying pathology.

## Conclusions

Our study is the first to provide insight into the initiating trigger of early phenotypic changes, which predispose cells of the injured proximal tubule to tubular injury. Elevated levels of TGF-β1 increase Cx43 hemichannel mediated ATP release, an effect which drives P2X7 mediated phenotypic changes linked to initiation of EMT in the tubular region of the kidney. With previous work in the Cx43^+/−^ UUO model linking reduced Cx43 expression to diminished levels of extracellular matrix deposition, fibroblast activation and macrophage infiltration [24], the current study utilises Peptide 5 and confirms that the protective effects as observed in the Cx43^+/−^ UUO mouse, appear to stem from inhibition of aberrant Cx43 mediated hemichannel ATP release. In conclusion, Cx43 represents, a viable therapeutic intervention for tubular damage in late stage CKD via restoration of the phenotypic and functional changes that culminate in an inflammatory and fibrotic phenotype.

## Data Availability

The datasets used and/or analysed during the current study are available from the corresponding author on reasonable request.

## Abbreviations

ATP: Adenosine triphosphate
AJ: Adherens junction
CKD: Chronic Kidney Disease
Cx43: Connexin43
HK2: Clonal tubular epithelial cells
ECAD: E-cadherin
GJIC: Gap junction mediated intercellular communication
hPTECS: Human primary proximal tubule epithelial cells
NCAD: N-cadherin
TGF-β1: Transforming Growth Factor
TJ: Tight Junction
TIF: Tubulointerstitial Fibrosis
UUO: Unilateral ureteral obstruction
Z0–1: Zona-Occludin
